# Genetic diversity and population differentiation of *Parrotia subaequalis* revealed by RAD-seq

**DOI:** 10.3389/fpls.2026.1724975

**Published:** 2026-03-02

**Authors:** Yang Yanfang, Zhai Wei, Chen Zijie, Du Yu, Lin Shu, Zhao Kai

**Affiliations:** 1 Provincial Key Laboratory of Biodiversity Research and Ecological Protection in Southwest Anhui Province, College of Life Sciences, Anqing Normal University, Anqing, China; 2 The Belt and Road Model International Science and Technology Cooperation Base for Biodiversity Conservation and Utilization In Basins of Anhui Province, Anqing, China; 3 College of Life Sciences, Shaanxi Normal University, Shaanxi, China

**Keywords:** conservation genetics, genetic diversity, *P. subaequalis*, phylogeographic structure, population genetics, SNP markers

## Abstract

*Parrotia subaequalis* (H. T. Chang) R.M. Hao & H.T. Wei is an endemic species of China that occupies an important phylogenetic position within the family Hamamelidaceae. To systematically evaluate its genetic diversity and population genetic structure, we conducted reduced-representation genome resequencing of 233 individuals from nine natural populations. In addition, Oxford Nanopore Technologies (ONT) long-read sequencing data and raw Hi-C data released in the NCBI public database were used to perform *de novo* genome assembly and chromosome-level scaffolding, generating a reference genome for population genetic analyses. A total of 2,716,976 SNPs were detected, and after quality control and filtering, 136,252 high-quality SNPs were retained for subsequent analyses. Genetic diversity analyses showed that the mean number of alleles (Na) across populations was 1.83, the effective number of alleles (Ne) was 1.24, the polymorphism information content (PIC) was 0.146, and nucleotide diversity (Pi) was 0.000037. The overall observed heterozygosity (Ho) and expected heterozygosity (He) were 0.092 and 0.170, respectively, indicating a moderately low level of genetic diversity in this species. Principal component analysis (PCA), population structure inference, and phylogenetic reconstruction based on SNP data consistently supported a distinct three-cluster genetic structure: the Taoling (TL) population formed a relatively independent genetic lineage; the Longchishan (LCS) and Gujingyuan (SDG) populations showed genetic proximity with partial overlap; and the remaining six populations clustered into another group. Pairwise *F*_st_ values among populations ranged from 0.0168 to 0.1082, indicating weak to moderate genetic differentiation among populations, with the highest differentiation observed between the TL population and the others. Linkage disequilibrium (LD) decay rates differed among populations, with slower LD decay in the LWS population, potentially associated with changes in its effective population size (Ne). Overall, this study revealed the geographic genetic structure and spatially restricted gene flow of *P. subaequalis*, providing important genetic evidence for defining conservation units and formulating *in situ* and *ex situ* conservation strategies, as well as for the preservation and utilization of germplasm resources.

## 1 Introduction

*Parrotia subaequalis* (H. T. Chang) R.M. Hao & H.T. Wei is a highly representative East Asian Tertiary relict tree species within the family Hamamelidaceae (Li et al., 1997; Deng et al., 1997). As one of only two extant species of the genus *Parrotia*—the other being the Iranian ironwood *Parrotia persica* (DC.) C.A.Mey.—*P. subaequalis* occupies a prominent position in phylogenetic studies of Hamamelidaceae and in research on relict lineages because of its unique phylogenetic status and its significance as an East Asian Tertiary relict species (Geng et al., 2015). Owing to its striking autumn foliage coloration, elegant crown architecture, and characteristic exfoliating bark, *P. subaequalis* is regarded as an ornamental horticultural tree species. Notably, its aboveground tissues—including leaves, stems, and insect-induced galls—are rich in diverse bioactive secondary metabolites (Wu et al., 1998). Tannins are predominant in the leaves, whereas the galls are characterized by benzofuran- and dibenzofuran-type phytoalexins, highlighting its potential and distinctive medicinal and scientific value beyond ornamental applications (Liu et al., 2020; Zhou et al., 2025).

In recent years, under increasing human disturbance and intensified habitat degradation and fragmentation, coupled with the species’ reliance on vegetative sprouting and insufficient sexual recruitment, natural regeneration of wild populations has become difficult, ultimately leading to a significant decline in population size (Yang Y. et al., 2025), and the species has been listed as Critically Endangered (CR) on the IUCN Red List (Geng et al., 2015). Current records show that wild *P. subaequalis* is mainly distributed in the low- to mid-elevation mountainous regions of Anhui, Jiangsu, and Zhejiang provinces, exhibiting a fragmented and discontinuous distribution pattern (Liu et al., 2021; Zhang et al., 2025). The southern slopes of the Dabie Mountains in Anhui, particularly the area from Huangwei in Yuexi County to Tangchi in Shucheng County, represent the core distribution region, where populations are most concentrated, abundant, and structurally intact. However, in the Shucheng and Yuexi areas on the southern slope of the Dabie Mountains, *P. subaequalis* populations are generally well preserved, and in some localities thousands of mature trees can still be observed forming continuous patches (Yan and Zhang, 2022). In contrast, populations in Tongcheng, which is closer to the mountain margins, have been severely affected by anthropogenic disturbance and are mostly characterized by small population sizes and highly fragmented, spatially scattered remnants. The level of genetic diversity and the genetic structure of populations in endangered plants constitute an important scientific basis for evaluating their survival potential and formulating effective conservation strategies (Ellstrand and Elam, 1993; Willi et al., 2022), as they not only reflect a species’ adaptive capacity to environmental change but are also directly linked to its long-term evolutionary potential and natural recovery ability (Pavlova et al., 2017). In species experiencing severe habitat fragmentation and pronounced geographic isolation, reductions in effective population size often lead to intensified genetic drift, increased risks of inbreeding, and decreased adaptive potential, thereby accelerating the process of endangerment (Young et al., 1996; Matesanz et al., 2017; Pavlova et al., 2017). Previous studies, based on morphological traits as well as multiple molecular markers—including nuclear DNA markers, such as inter-simple sequence repeat (ISSR) and nuclear genes, and chloroplast DNA (cpDNA) markers—have revealed pronounced genetic differentiation among *P. subaequalis* populations across different geographic regions, and have generally considered geographic isolation and population fragmentation to be the primary drivers of reduced genetic diversity and population divergence (Li and Zhang, 2015). The limited long-distance dispersal ability of seeds in *P. subaequalis* allows geographic barriers to persistently restrict gene flow over long temporal scales. In recent years, with the application of chloroplast genome data and approaches such as ecological niche modeling, the phylogeographic pattern of *P. subaequalis* has been further explored (Yan and Zhang, 2022). However, these studies remain constrained to varying degrees by limited sample sizes, insufficient spatial coverage of sampling, and the resolution of molecular markers, and a systematic and comprehensive understanding of the overall genetic structure and potential adaptive mechanisms of its wild populations across the entire distribution range is still lacking (Geng et al., 2015; Abdelaal et al., 2019; Yun-xia et al., 2020). Against the backdrop of ongoing global climate change and intensifying habitat fragmentation, *P. subaequalis*, a narrowly distributed Tertiary relict tree species, is facing increasingly severe survival pressures, particularly in its main distribution areas—the Dabie Mountains and their southern and northern slopes, as well as the two major concentration centers south and north of the Yangtze River (Geng et al., 2015; Zhang et al., 2022).

In recent years, the rapid development of genomics has provided new approaches for studying the population genetics of endangered plants (Tian and Ma, 2021; Theissinger et al., 2023). Reduced-representation genome sequencing technologies, such as restriction site–associated DNA sequencing (RAD-seq), genotyping-by-sequencing (GBS), and specific-locus amplified fragment sequencing (SLAF-seq), allow the identification of large numbers of genome-wide single nucleotide polymorphisms (SNPs), thereby enabling high-resolution characterization of genetic variation and population structure (Andrews et al., 2016). SNP-based approaches—including population structure analysis, principal component analysis (PCA), and phylogenetic tree construction have been widely applied in plant genetics (Zhao, 2023).

Based on this, the present study employed reduced-representation genome sequencing to analyze 233 samples from nine natural populations of *P. subaequalis* distributed across Anhui Province and adjacent regions. Through analyses including PCA, population structure inference, phylogenetic reconstruction, fixation index (*F*_st_), nucleotide diversity (Pi), and linkage disequilibrium (LD) decay (Kardos et al., 2021), the study aimed to: (1) assess the level of genetic diversity within natural populations of *P. subaequalis*; (2) reveal the genetic structure and geographical differentiation among populations; and (3) explore the roles of geographic isolation and historical processes in shaping population divergence. The results will provide a genetic basis for understanding the mechanisms underlying the endangerment of *P. subaequalis*, and offer theoretical guidance for its *in situ* conservation, *ex situ* preservation, and population restoration. Furthermore, this research will provide valuable insights for conservation genetics studies of other relict and endangered plant species in East Asia.

## 2 Materials and methods

### 2.1 Plant material and DNA extraction

**Figure 1:**
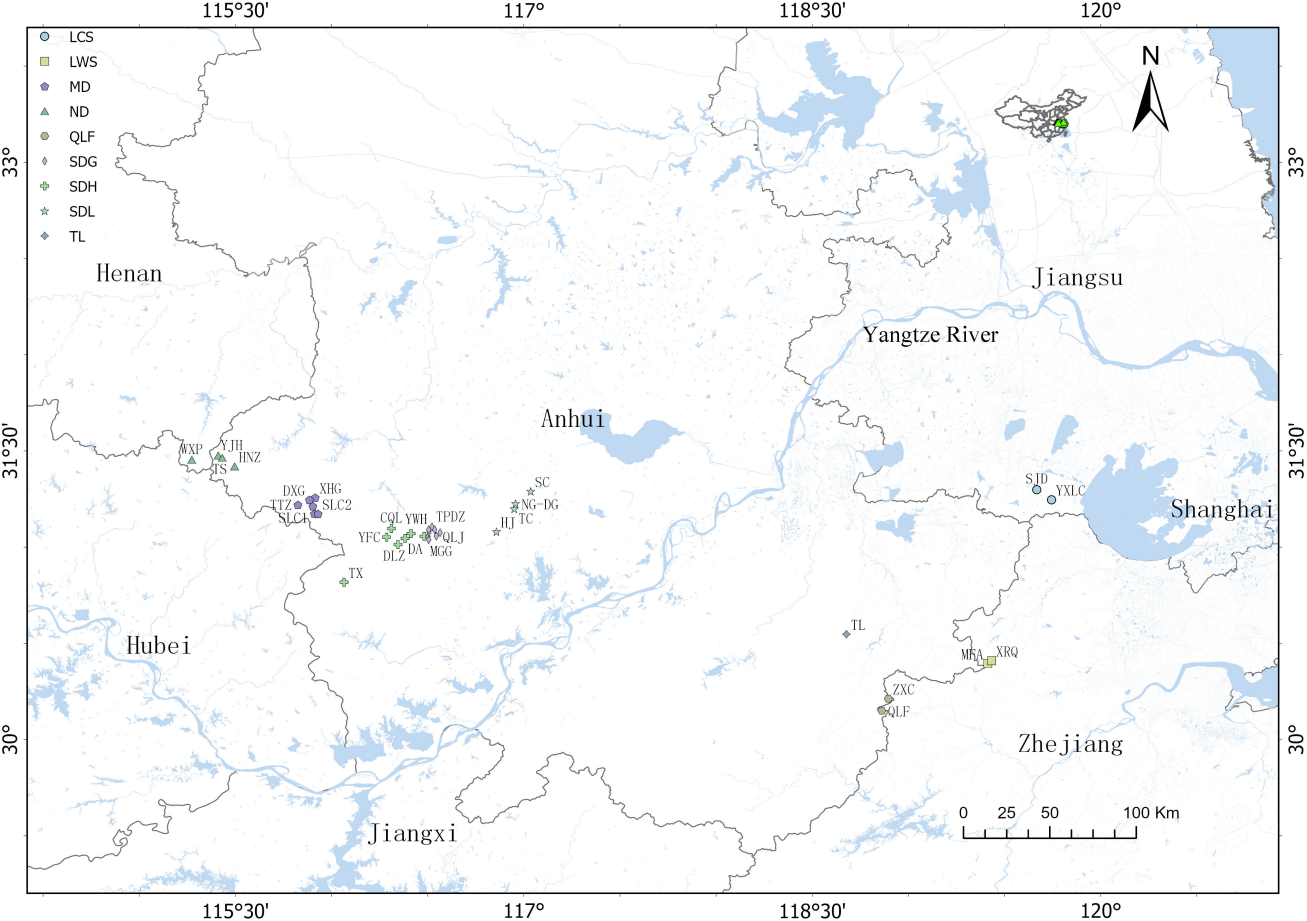
Geographic distribution of *P. subaequalis*. Sampling locations are indicated by symbols of different shapes and colors, and uppercase abbreviations correspond to site names; full names are provided in **Table 1**.

A total of 233 species/cultivars were sampled (**Figure 1**, **Table 1**). Fresh leaf tissue (≈100 mg) was ground in liquid nitrogen, and genomic DNA was isolated using a modified CTAB protocol (Doyle & Doyle, 1987). DNA concentration and integrity were assessed with a Qubit 4 fluorometer (Thermo Fisher Scientific, Waltham, MA, USA) and 1% agarose gel electrophoresis, respectively. A minimum of 1.5 µg DNA per sample (OD_260_/OD_280_ = 1.8–2.0) was used for library construction.

**Table 1:** Sampling information of *P. subaequalis* populations.

Population code	Population size	Sampling site (location)	Longitude (E)	Latitude (N)
ND (Northern Dabie Mountains), 30 individuals	Medium, 219 individuals	WXP (waxiping)	115.273333	31.456389
		YJH (yangjiahe)	115.410856	31.478778
		TS (tuanshu)	115.431603	31.467177
		HNZ (huaniangzhai)	115.495775	31.422333
MD (Central Dabie Mountains), 33 individuals	Large, 1263 individuals	TTZ (tiantangzhai)	115.825411	31.221165
		XHG (xiaohegu)	115.915068	31.258577
		DXG (daixiagu)	115.885278	31.246944
		JCY (jiucaiyan)	115.903130	31.213803
		SLC1 (shenglicun1)	115.911192	31.174113
		SLC2 (shenglicun2)	115.930172	31.173959
SDH (Southern Dabie Mountains; Huangwei), 31 individuals	Large, 2131 individuals	CQL (cheqiling)	116.311263	31.096848
		YFC (yunfengcun)	116.286020	31.052247
		DLZ (dalongzu)	116.345910	31.013605
		DA (daao)	116.382290	31.045244
		DLSK (dinglingshuiku)	116.413577	31.070715
		TX (tianxia)	116.065143	30.817908
SDG (Southern Dabie Mountains; Gujingyuan), 30 individuals	Large, 1024 individuals	DXC (daxiecun)	116.481070	31.056004
		XW (xiawan)	116.501775	31.054062
		MGG (muguacun)	116.505454	31.038113
		LWC (longwangcun)	116.506609	31.077950
		YWH (yangwanhe)	116.505540	31.090783
		TPDZ (taipingdiazhan)	116.522642	31.105181
		LW (langwo)	116.536504	31.090776
		ZJC (zhaojiaochong)	116.544814	31.057475
		QLJ (qingliangjian)	116.564805	31.075172
SDL (Southern Dabie Mountains; Longmianshan), 32 individuals	Large, 2869 individuals	HJ (huangjia)	116.857836	31.080206
		TC (tangchi)	116.949917	31.198604
		NG-DG (nangang-daguan)	116.957115	31.226172
		SC (shucha)	117.035905	31.290054
TL (Taoling), 12 individuals	Small, 92 individuals	TL (taoling)	118.677692	30.547642
QLF (Qingliangfeng), 25 individuals	Medium, 242 individuals	ZXC (zhuxiancun)	118.895217	30.212849
		QLF (qingliangfeng)	118.861663	30.150156
LWS (Longwang Mountain), 11 individuals	Medium, 330 individuals	MFA (mafengan)	119.412644	30.395567
		XRQ (xianrenqiao)	119.432516	30.409585
LCS (Longchi Mountain), 29 individuals	Medium, 202 individuals	YXLC (yixinglinchang)	119.744252	31.246149
		SJD (shanjuandong)	119.667393	31.298473

Population size categories: Large (≥ 1000 individuals), Medium (200–999 individuals), and Small (< 200 individuals).

### 2.2 RAD-seq library preparation and Illumina sequencing

Paired-end RAD-seq libraries with an average insert size of approximately 350 bp were constructed using a single-enzyme (*EcoRI*) digestion strategy. A total of 233 individuals of *P. subaequalis* were included in the analysis. Sequencing depth was determined based on the estimated genome size (~1 Gb), with one individual sequenced (HW215a) at higher depth and the remaining individuals sequenced at moderate depth. Library preparation was carried out using the TruSeq Nano DNA HT Sample Prep Kit (Illumina, San Diego, CA, USA). Genomic DNA was fragmented to an average size of ~350 bp using a Covaris M220 ultrasonicator (Covaris, Woburn, MA, USA). Fragmented DNA was subsequently subjected to end repair, A-tailing, and adapter ligation, followed by PCR enrichment (eight cycles) and purification using AMPure XP beads (Beckman Coulter, Brea, CA, USA).

Library fragment size distribution (main peak 300–500 bp) was evaluated using an Agilent 2100 Bioanalyzer (Agilent Technologies, Santa Clara, CA, USA), and library concentrations were determined by quantitative PCR. High-throughput sequencing was performed on an Illumina NovaSeq 6000 platform (Illumina, San Diego, CA, USA) at Novogene (Beijing, China), generating 150 bp paired-end reads.

### 2.3 Quality control and read filtering

Raw reads were processed with fastp v0.20.0 (Chen et al., 2018) to remove (i) reads containing ≥10% ambiguous bases (N), (ii) reads with >10 nt aligned to adapters (≤10% mismatches), (iii) reads where ≥50% bases had Phred quality <5, and (iv) PCR duplicates.

### 2.4 Read alignment and SNP calling

The sequencing data have been deposited in the China National GeneBank DataBase (CNGBdb) under accession number CRA033599. Filtered clean reads were aligned to the HW215a reference genome using BWA-MEM v0.7.17 (Li, 2013) with default parameters. The reference genome was assembled in this study based on publicly available Oxford Nanopore Technologies (ONT, SRR23215159) long-read data and Hi-C (SRR23215049) data downloaded from NCBI. Initial genome assembly was performed using NextDenovo, followed by Hi-C–assisted chromosome anchoring using HapHiC, resulting in a chromosome-level genome assembly.

Alignment results were sorted and converted to BAM format using SAMtools v1.9 (Li et al., 2009), and PCR duplicate reads were identified and marked using Picard v2.18.11. SNPs were called for all individuals using SAMtools mpileup and the Bayesian approach implemented in bcftools v1.9, with the following parameters: -q 1 -C 50 -t SP,DP -m 2 -F 0.002. Variant sites were subsequently subjected to hard filtering with the following criteria: sequencing depth between 3 and 50, root mean square (RMS) mapping quality ≥ 20, minor allele frequency (MAF) ≥ 0.05, and missing rate ≤ 10%.

### 2.5 Population structure and phylogenetic analyses

Multiple complementary approaches were employed to analyze population genetic structure and phylogenetic relationships, including PCA, phylogenetic tree reconstruction, population structure inference, and population genetic statistics.

Based on the filtered SNP dataset, PCA was performed using PLINK v1.9 (Chang et al., 2015), and genetic structure among samples was visualized using the first two principal components (PC1 and PC2). The processing and visualization of PCA results were carried out using custom scripts.

For phylogenetic analysis, pairwise genetic distance matrices among individuals were first calculated using PLINK v1.9. The distance data were then formatted using custom scripts, and phylogenetic trees were constructed in MEGA11 (Tamura et al., 2021) using the neighbor-joining (NJ) method.

Population genetic structure was inferred using ADMIXTURE v1.23 (Alexander et al., 2009), with the number of ancestral populations (K) set from 2 to 8 to estimate individual ancestry proportions. The post-processing and visualization of ADMIXTURE results were performed using custom scripts.

Population genetic diversity and differentiation were assessed using nucleotide diversity (π) and pairwise *F*_st_ statistics. These metrics were calculated using VCFtools with a sliding-window approach, employing a window size of 10 kb and a step size of 5 kb. Visualization of π and *F*_st_ results was also conducted using custom scripts.

LD decay analysis was conducted using PopLDdecay, which directly calculates pairwise linkage disequilibrium (r²) from VCF files and summarizes the relationship between r² and physical distance to infer genome-wide LD decay, without employing sliding windows (Zhang et al., 2019).

All data processing, result summarization, and figure generation were carried out in the R v4.2.1 environment (R Core Team, 2024).

## 3 Results

### 3.1 Sequencing data quality

The genomic region, excluding gaps, measures 146,350,253 bp, with an average alignment rate of 81.87% across the population samples (**Supplementary Table 1**.). The average sequencing depth for the genome (excluding gaps) is 20.77x, based on reads with an alignment quality greater than 0. SNP detection was conducted using tools such as SAMTOOLS, identifying a total of 2,716,976 SNP sites. After applying stringent filtering criteria—including a minimum sequencing depth of 3 for each sample, a missing data rate below 30%, and a MAF greater than 0.01—136,252 high-quality SNPs were retained for further analysis.

### 3.2 Population genetic diversity analysis

#### 3.2.1 Population genetic structure analysis

**Figure 2:**
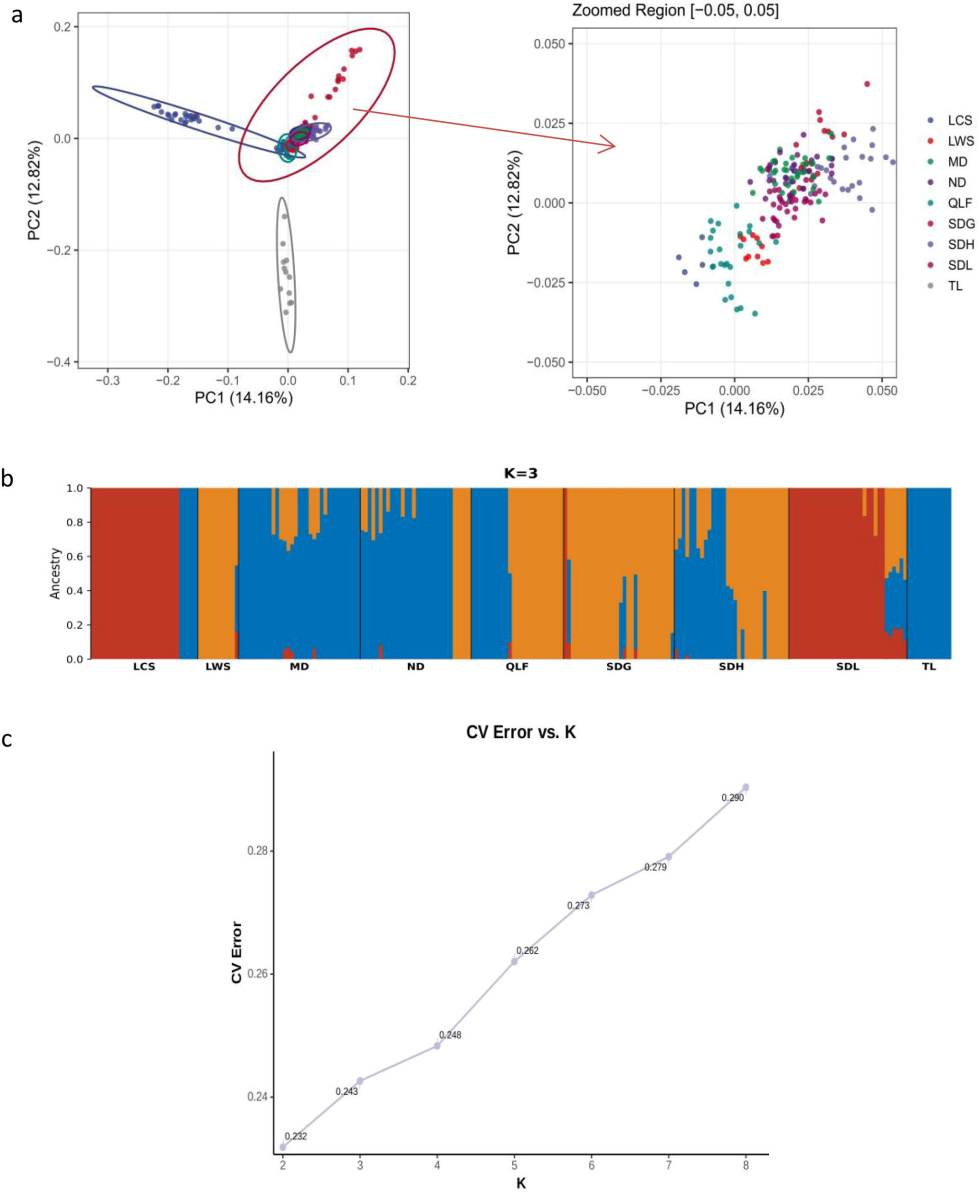
Principal component analysis (PCA) and Population Structure of *P. subaequalis* Populations **(a)** Principal Component Analysis (PCA) showing the distribution of different *P. subaequalis* populations in the principal component space; **(b)** Population structure of *P. subaequalis* as inferred by ADMIXTURE analysis. **(c)** CVerror vs K.

We used PCA and population genetic structure analysis to reveal the genetic differentiation of *P. subaequalis* populations. The PCA analysis showed that the populations could be broadly divided into three major groups in the principal component space (**Figure 2a**). Specifically, the TL population clustered independently in the PCA plot and was clearly separated from all other populations, indicating pronounced genetic differentiation between TL and the remaining populations. The LCS population formed a relatively distinct cluster in the PC1-negative and PC2-positive region of the PCA space, showing limited overlap with other populations. In contrast, populations from the Dabie Mountains region, including MD, ND, QLF, SDG, SDH, SDL, and LWS, were mainly distributed in the central region of the PCA space with substantial overlap, suggesting relatively weak genetic differentiation among these populations.

To further investigate population genetic structure, we conducted ADMIXTURE analyses (**Figure 2b**). In the RAD-seq dataset used in this study, the cross-validation (CV) error showed overall limited ability to discriminate among different K values and did not provide strong statistical support for any single K (**Figure 2c**). In particular, the difference in CV error between K = 2 and K = 3 was small, and no clear inflection point was observed in the CV error curve. In combination with the clustering patterns revealed by PCA, K = 3 was selected for further presentation and discussion based on its greater biological interpretability, rather than being considered the statistically optimal model. At K = 3, the ADMIXTURE results revealed a relatively clear pattern of ancestral component differentiation (**Figure 2b**): the TL population was almost entirely composed of a single ancestral component, indicating a distinct genetic composition, whereas the LCS, SDG, and QLF populations exhibited varying degrees of admixture, suggesting more complex genetic backgrounds.

#### 3.2.2 Genetic differentiation among populations and conservation prioritization

**Figure 3:**
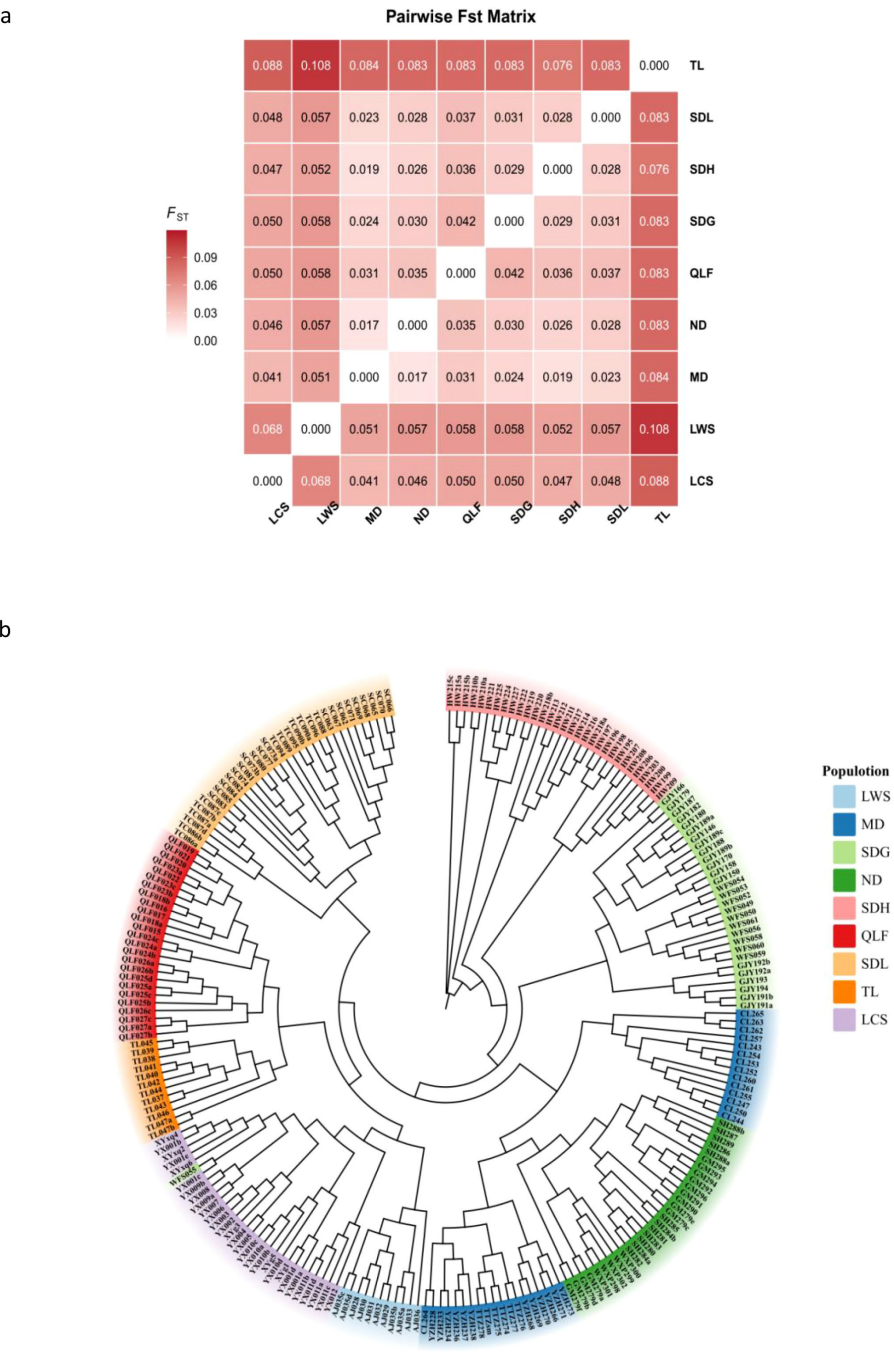
Pairwise *F*_st_ matrix and unrooted neighbor-joining (NJ) phylogenetic relationships among *P. subaequalis* populations. **(a)** Heatmap showing pairwise *F*_st_ values among populations. Population names are shown on both axes, and each cell displays the *F*_st_ value for a given population pair, with color intensity corresponding to the magnitude of the value as indicated by the color scale. **(b)** Neighbor-joining (NJ) tree constructed based on genome-wide SNP data. Tips are colored according to population identity.

Pairwise *F*_st_ analysis (**Figure 3a**) revealed pronounced differences in genetic differentiation among populations. The TL population exhibited consistently higher *F*_st_ values in comparisons with all other populations, ranging from 0.076 to 0.108, with the highest level of differentiation observed between TL and the LWS population (*F*_st_ = 0.108). This pattern indicates that TL shows a stable and consistent genetic separation from the remaining populations. In contrast, populations from the Dabie Mountains region (ND, MD, SDG, SDH, and SDL) displayed overall low levels of genetic differentiation, with pairwise *F*_st_ values mainly distributed within the range of 0–0.058, suggesting relatively weak differentiation among these populations. The LCS, QLF, and LWS populations exhibited intermediate levels of differentiation, with *F*_st_ values of 0.041–0.068 in comparisons with the Dabie Mountains populations, while their differentiation from TL was markedly higher (0.083–0.088), reflecting an intermediate pattern of genetic divergence.

The neighbor-joining (NJ) phylogenetic tree constructed from genome-wide SNPs (**Figure 3b**; **Supplementary Figure 1**) was highly consistent with the *F*_st_ results. The tree showed that individuals from the TL population clustered into a well-supported and clearly separated clade, indicating a high degree of genetic coherence within this population. In contrast, populations from the Dabie Mountains region formed a closely clustered assemblage, with substantial intermixing of lineages among populations, consistent with their generally low pairwise *F*_st_ values (mostly < 0.05). The LCS, QLF, and LWS populations occupied phylogenetic positions intermediate between the TL population and the Dabie Mountains populations; their individuals did not form mixed clades with TL but instead showed closer phylogenetic affinities to the Dabie Mountains populations.

#### 3.2.3 Genetic diversity and population evolution

**Figure 4:**
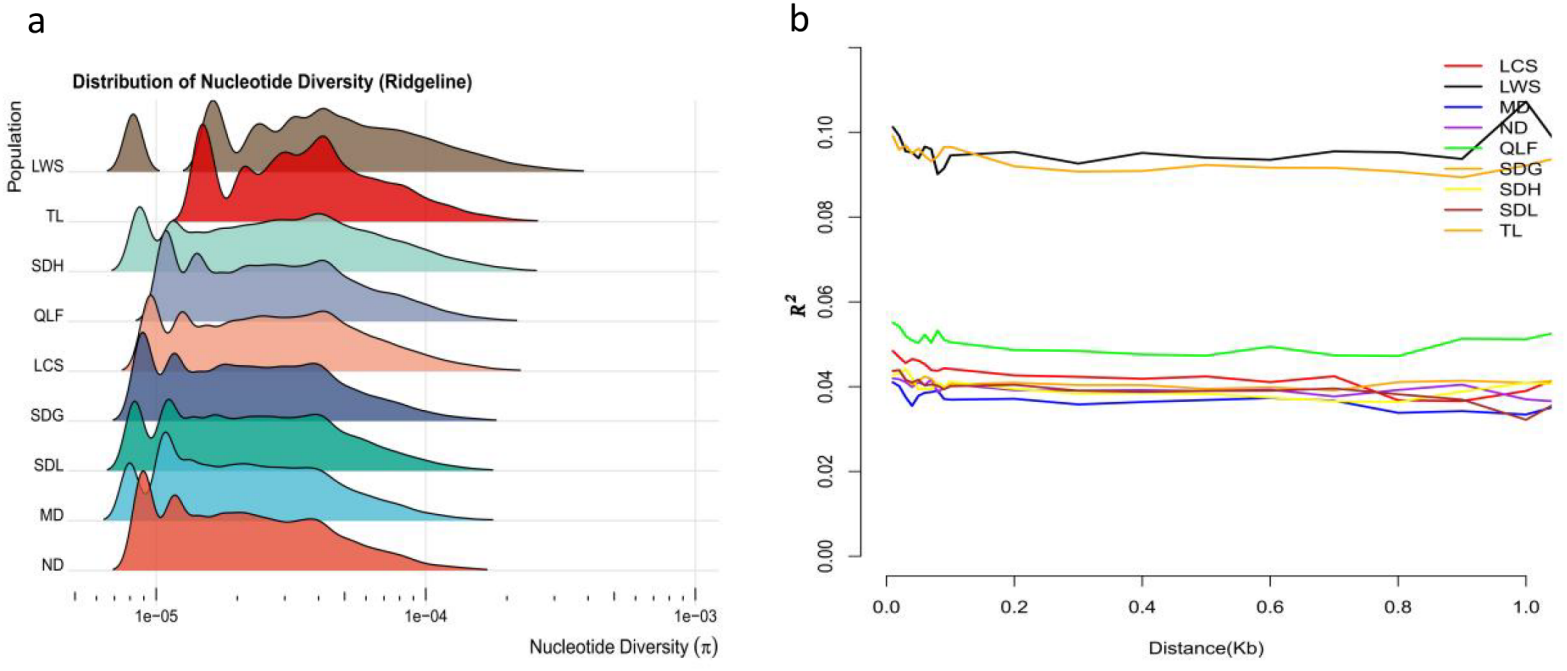
Nucleotide diversity and linkage disequilibrium patterns of *P. subaequalis* populations inferred from genome-wide SNP data. **(a)** Distribution of nucleotide diversity (π) across nine populations (LCS, LWS, MD, ND, QLF, SDG, SDH, SDL, and TL), visualized as ridgeline density plots showing the distribution of π values across loci for each population. **(b)** Linkage disequilibrium (LD) decay patterns showing the relationship between LD (measured as r²) and physical distance (kb) between SNP pairs for each population.

Nucleotide diversity (Pi), representing population genetic diversity, showed pronounced differences among populations in the ridgeline distribution plot (**Figure 4a**, **Table 2**). The shape and dispersion of Pi distributions varied markedly among populations, with Pi values overall occurring at the 10^-5^ magnitude (approximately 2.76 × 10^-5^–5.58 × 10^-5^). The LWS population exhibited the widest Pi distribution with a pronounced right tail, indicating a broad range of genetic diversity across genomic loci. In contrast, populations from the Dabie Mountains region (ND, MD, SDG, SDH, and SDL) showed more concentrated Pi distributions, with peaks shifted toward lower values, suggesting relatively homogeneous levels of genetic diversity. The TL population displayed an intermediate Pi distribution, broader than those of the Dabie Mountains populations but narrower than that of the LWS population, while LCS and QLF populations also exhibited intermediate Pi distribution patterns, overlapping to varying extents with both groups.

**Table 2:** Genetic diversity parameters of *P. subaequalis* populations.

Group	Na	Ne	Ho	He	PIC	Pi
LCS	1.828747	1.242648	0.102638	0.168898	0.145118	0.0000361
LWS	1.656235	1.253784	0.112475	0.166128	0.139332	0.0000558
MD	1.944095	1.236279	0.084333	0.172040	0.150417	0.0000284
ND	1.897704	1.232613	0.077628	0.167308	0.145636	0.0000276
QLF	1.855402	1.247426	0.093549	0.173390	0.149359	0.0000359
SDG	1.898490	1.239458	0.076094	0.170732	0.148103	0.0000298
SDH	1.933323	1.254138	0.106768	0.181629	0.157605	0.0000403
SDL	1.916260	1.243819	0.089045	0.174489	0.151541	0.0000302
TL	1.544661	1.240410	0.088903	0.151926	0.125612	0.0000455

Linkage disequilibrium (LD) decay analysis (**Figure 4b**) further revealed differences in population genetic structure. In all populations, LD (measured as r²) declined with increasing physical distance, indicating a general genome-wide decay of linkage. However, the extent and rate of LD decay differed substantially among populations. The LWS population maintained higher r² values across genomic distances and exhibited the slowest LD decay, whereas populations from the Dabie Mountains region showed lower overall r² levels and more rapid LD decay, with largely similar decay trajectories. The TL population again displayed an intermediate LD decay pattern, distinct from both the LWS population and the Dabie Mountains populations.

Taken together, the Pi distribution characteristics and LD decay patterns indicate clear differences in genetic diversity and linkage structure among populations of *P. subaequalis*, with LWS and TL populations differing markedly from the Dabie Mountains populations.

### 3.3 Relationship between geographic distribution and genetic structure

Populations from the Dabie Mountains region (ND, MD, and SD) are mainly distributed north of the Yangtze River and show a spatially continuous distribution, forming a relatively compact geographic unit. In contrast, populations located south of the Yangtze River and further east (LCS, TL, LWS, and QLF) are spatially separated from the Dabie Mountains populations and exhibit a more fragmented distribution pattern.

Consistent with the genetic structure analyses, Dabie Mountains populations cluster tightly in genetic space, indicating relatively weak population differentiation. In contrast, the TL population, located at the southern margin of the species’ distribution, is clearly separated from other populations in the PCA and shows a pronounced shift along the principal component axis, reflecting its distinct genetic structure. Meanwhile, the LCS population shows the greatest deviation from the Dabie Mountains populations in genetic space, whereas the LWS and QLF populations occupy intermediate positions. Overall, genetic differentiation among *P. subaequalis* populations shows a clear spatial correspondence with the Yangtze River system and regional mountain distributions.

## 4 Discussion

Compared with traditional dominant markers or low-locus codominant markers, which are often constrained by limited marker numbers and restricted genome coverage in small and strongly structured populations, RAD-seq approaches can generate high-density SNP datasets, thereby substantially improving the accuracy of genetic structure inference, population differentiation, and demographic history reconstruction (Andrews et al., 2016; Cano et al., 2022). Accordingly, we conducted RAD-seq analyses on 233 individuals sampled from nine natural populations. Comparisons with previous ISSR and SSR/cpSSR studies reveal a high degree of concordance across marker systems, consistently supporting the general conclusion of strong interpopulation differentiation and limited gene flow in *P. subaequalis*. ISSR analyses reported a high GST (coefficient of gene differentiation) and a low Nm (number of migrants per generation), which were attributed to an “island-like” distribution driven by mountain isolation (Yun-xia et al., 2020), whereas SSR/cpSSR studies indicated that overall genetic diversity is not low but spatial structure is pronounced, with maternally inherited chloroplast markers typically showing stronger differentiation than nuclear loci (Zhang et al., 2018). The results demonstrate pronounced genetic differentiation across the distribution range of *P. subaequalis*, with clustering and ordination analyses consistently identifying three major genetic clusters across multiple algorithms, indicating long-term restriction of gene flow and clearly resolved lineage divergence. Notably, nucleotide diversity at the species level was moderate, whereas strong heterogeneity was evident at the population level (Yang R. et al., 2025). In particular, the TL population showed the lowest values of Na, He, and PIC, and exhibited a markedly slower decay of linkage disequilibrium (LD), suggesting stronger effects of genetic drift and the possible influence of historical bottlenecks or a persistently small effective population size (Waples, 2024). One scenario involves a long-isolated refugial lineage with persistently small effective population size, highlighting its irreplaceable evolutionary uniqueness; the other involves a population that was not historically extremely isolated but has undergone severe recent contraction due to habitat fragmentation and human disturbance, leading to the accumulation of inbreeding and drift signals and an increased risk of fitness decline. These alternative hypotheses could be further evaluated using whole-genome resequencing, demographic modeling, and genome–environment association analyses in future studies (Song et al., 2023).

At the mechanistic level, the combination of “strong structure with moderate but highly uneven diversity” observed in *P. subaequalis* likely arises from the coupling of population-level ecological processes, landscape resistance, and historical climatic events. Previous ecological studies have shown that populations are spatially aggregated at fine scales, exhibit pronounced variation in diameter-class structure (Li and Zhang, 2015). Such aggregation and small-population effects can increase local relatedness, reduce effective population size and effective recombination, thereby intensifying genetic drift and accelerating local loss of diversity, ultimately amplifying interpopulation differentiation under fragmented conditions (Young et al., 1996). At the landscape scale, complex mountainous topography, lowland isolation, and major river barriers jointly increase resistance to dispersal, consistent with landscape genetic theory predicting that geographic and environmental resistance shapes genetic variation at fine spatial scales (Cruzan and Hendrickson, 2020; Sandurska et al., 2024; Ko et al., 2024). Moreover, recent habitat suitability projections indicate that the current suitable habitat of *P. subaequalis* is spatially fragmented and may undergo range shifts under future climate scenarios (Yan and Zhang, 2022; Zhang et al., 2025; Zhou et al., 2025). Population-level ecological niche models further suggest that different populations may occupy distinct environmental spaces, implying spatial heterogeneity in vulnerability and conservation priority (Forester et al., 2018; Cruzan and Hendrickson, 2020). Together, these findings provide a basis for identifying potential long-term refugia and regions of elevated genetic risk. Under this spatial context, our results suggest that conservation and management actions should be differentially implemented at the lineage/population level. Overall, the TL population should be treated as a high-priority target, with emphasis on *in situ* conservation and habitat management to minimize genetic risks arising from ongoing disturbance and further isolation. In contrast, SDH and the MD, SDL, and QLF populations show relatively higher values across multiple diversity indices and are more likely to function as key reservoirs of species-wide genetic variation. These populations may therefore be regarded as potential long-term refugia, and conservation efforts should prioritize maintaining habitat integrity and population stability, while preserving or enhancing landscape connectivity where feasible to reduce increasing isolation and sustain long-term adaptive potential. Notably, the LWS population exhibits comparatively high nucleotide diversity (Pi), suggesting that it may retain distinctive genetic variation; thus, in addition to habitat protection, improvements in habitat quality and connectivity at the regional scale may facilitate the long-term maintenance of its genetic resources. For populations with intermediate diversity levels (e.g., ND, SDG, and LCS), conservation planning should consider the combined effects of topographic barriers and landscape resistance on dispersal and gene flow and aim to improve the potential for genetic exchange among populations while strengthening habitat protection.

*P. persica* is naturally distributed mainly within the Hyrcanian forest belt along the southern coast of the Caspian Sea (northern Iran and southeastern Azerbaijan), where suitable habitats are relatively continuous. In contrast, *P. subaequalis* has a narrow and disjunct, patchy distribution across montane regions of eastern China, with stronger population isolation, which likely facilitates pronounced interpopulation genetic differentiation (Wang et al., 2025; Zhang et al., 2025). Similarly, other endangered woody species within Hamamelidaceae, such as *Loropetalum subcordatum*, show extremely low within-population diversity and strong interpopulation differentiation, clearly warning that *ex situ* conservation efforts lacking representative sampling may fail to capture *in situ* genetic lineages (Gong et al., 2010; Li et al., 2018). Phylogeographic studies of multiple Tertiary relict tree species in the subtropical regions of central and eastern China further indicate that genetic clusters or haplotype lineages often correspond to mountain systems, reflecting the persistence of multiple refugia (Wang et al., 2009; Zhang et al., 2015; Zhang et al., 2025). In some narrowly distributed ancient endemics, a characteristic pattern of “moderate diversity, high inbreeding, and clearly defined clusters” is frequently observed (Zhang et al., 2021). Collectively, these patterns suggest that once connectivity is lost in narrowly distributed relict trees, genetic drift and isolation rapidly dominate the formation of genetic structure, and conservation assessments based solely on species-level mean metrics may underestimate risk, necessitating a shift toward lineage- or management-unit-based decision frameworks (Coates et al., 2018).

Overall, our study reveals that *P. subaequalis* harbors a moderate level of genetic diversity at the species level but exhibits a clear geographic genetic structure, with highly differentiated lineages and several distinct genetic units. This pattern indicates long-term restrictions on interpopulation gene flow and the formation of a stable lineage-divergence framework. Accordingly, our findings support a conservation framework based on population genetic characteristics: populations with reduced genetic variation and pronounced differentiation should be prioritized for risk mitigation and lineage preservation, with distinct lineages treated as independent management units for differentiated conservation actions; in contrast, populations with relatively higher genetic variation should be managed to maintain genetic diversity and enhance long-term adaptive potential. In addition, *ex situ* conservation efforts should adopt representative sampling strategies based on major genetic clusters to capture lineage-level diversity and regional genetic components of the species. Future studies integrating whole-genome resequencing, demographic modeling, and genome–environment association analyses will help further elucidate the mechanisms underlying lineage divergence and refine conservation strategies.

## Data Availability

The datasets presented in this study can be found in online repositories. The names of the repository/repositories and accession number(s) can be found in the article/**Supplementary Material**.
